# A Systematic Review Exploring Empirical Pharmacogenomics Research Within Global Indigenous Populations

**DOI:** 10.1002/mgg3.70018

**Published:** 2024-10-22

**Authors:** Bushra Farah Nasir, Ritwika Vinayagam, Luciana Massi, Shivashankar H. Nagaraj, Maree Toombs, Kym M. Rae

**Affiliations:** ^1^ Toowoomba Regional Clinical Unit, Medical School, Faculty of Medicine The University of Queensland Toowoomba Queensland Australia; ^2^ School of Public Health, Faculty of Medicine The University of Queensland Brisbane Queensland Australia; ^3^ Mater Research Institute The University of Queensland Brisbane Queensland Australia; ^4^ Centre for Genomics and Personalised Health and the School of Biomedical Sciences Queensland University of Technology Brisbane Queensland Australia; ^5^ Translational Research Institute Queensland University of Technology Brisbane Queensland Australia; ^6^ School of Population Health, Faculty of Medicine and Health The University of New South Wales Sydney New South Wales Australia

**Keywords:** genomics, indigenous genomics research, pharmacogenomics

## Abstract

**Background:**

This systematic review aims to highlight the scope of pharmacogenomics research within global Indigenous populations. This review also explores the barriers and facilitators of pharmacogenomics research within this population.

**Methodology:**

A systematic review of literature was conducted to identify and present an understanding of current empirical evidence demonstrating the conduct of genomics or pharmacogenomics research within global Indigenous populations (PROSPERO registration: CRD42021257226). Using key search terms, relevant databases were searched for articles published between January 2010 and July 2022. Screening, data extraction, and analysis was conducted using well‐defined inclusion criteria. Quality assessment and risk of bias appraisal was conducted using the mixed methods appraisal tool. Indigenous community engagement and participation in genomics research was assessed using the social‐ecological framework.

**Results:**

From the 427 articles identified, 77 articles met inclusion criteria and underwent full‐text screening. Of these, 30 articles were included in the final review, with 16 being quantitative and 14 either qualitative or mixed methods studies. Most studies were conducted with native Indigenous populations from the United States of America (36%). Content analysis revealed that studies either explored genetic variations associated with disease in Indigenous populations (23%) or markers for drug metabolism (30%) or were designed to understand perspectives of genomics research within this population (47%). Perspectives included the exploration of the role of participants in research, benefits or outcomes achieved from participation in genomics research, and levels of Indigenous engagement and participation in genomics research.

**Conclusions:**

This review highlights a growing gap in Indigenous genomics research globally. It presents several important considerations from Indigenous participants, identifying how researchers can co‐create culturally safe and inclusive design, implementation, analysis, and subsequent outcomes of genomics research involving Indigenous people. Indigenous governance, self‐determination and leadership is essential, with researchers required to be responsive to such fundamental partnerships for research to progress.

## Graphical abstract

This systematic review aims to highlight the scope of pharmacogenomics research within global Indigenous populations. This review also explores the barriers and facilitators of pharmacogenomics research within this population. It presents several important considerations from Indigenous participants, identifying how researchers can co‐create culturally safe and inclusive design, implementation, analysis, and subsequent outcomes of genomics research involving Indigenous people. Indigenous governance, self‐determination and leadership is essential, with researchers required to be responsive to such fundamental partnerships for research to progress.

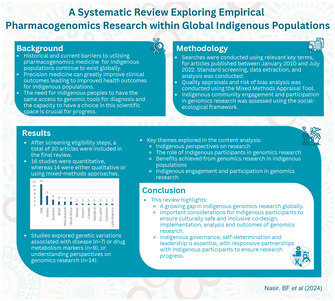

## 1 Introduction

Precision medicine has generated considerable hope of beneficial clinical outcomes in its scope and its potential to transform medicine and healthcare, ultimately improving population health (Bayer and Galea 2015; Collins and Varmus 2015). A steady stream of new discoveries linking genes and single‐nucleotide polymorphisms (SNPs) to disease risk or drug responses have paved the way for advances in genomic medicine. However, despite this wealth of genomic knowledge and consequent clinical benefits, equitable clinical research, provision of genomic treatment services and culturally safe, acceptable genomic diagnostic tools, the treatment options for global Indigenous populations have remained limited. The ability to harness precision medicine approaches to address ongoing health disparities within Indigenous populations is needed, but it requires research using culturally respectful approaches with Indigenous guidance to select appropriate methods to understand the true nature of the genetic variation of Indigenous populations. The diversity of ethnicity, race, and ancestry in the way that genetic knowledge is discovered, classified, and applied considerably limits efforts to achieve health equity and eliminate health disparities for Indigenous people. The multidimensional nature of a person's identity, life experiences, and exposure to social determinants of health are not reflected within most large national genomics datasets, thus further limiting efforts to advance equitable genomic research (Landry et al. 2018; Bonham, Green, and Pérez‐Stable 2018). Inadequate sampling and representation of genetic diversity are widely recognized as a significant bias inherent in genomic databases, which has direct implications for the healthcare available to minority populations (Perera 2019). There are concerns that those who continue to experience the greatest health disparities are benefitting the least from these progressive scientific discoveries. Thus, the challenge is to ensure that the option to engage in genomic research and clinical care is rightfully distributed among many population groups, and that these databases and clinical testing consider genetic diversity to achieve meaningful outcomes. The ability of Indigenous peoples to have the same access to genomic tools for diagnosis and the capacity to have a choice in this scientific space is therefore crucial.

In this era of precision medicine, genetic variation is used to predict drug responses, translating genomic medicine into direct applicability in real‐time patient care through a sophisticated “bench‐to‐bedside” pathway (Singh 2020). The scope for health improvement using medical advancements such as pharmacogenomics, which is the study of DNA variations to develop personalized treatment approaches (Roden et al. 2019) through the analysis of genetically distinct populations (Nagaraj and Toombs 2021) is becoming increasingly advanced. Even so, the possibility of exacerbating existing inequities and health disparities that Indigenous people experience is a striking reality, with a glaring “genomic gap” (Jaya Shankar et al. 2022) becoming increasingly evident. As personalized medicine and treatment focus attention on prevention research and the exploration of targeted therapies, pharmaceutical options, and public health strategies (Singh 2020), it is vital that efforts be made to improve the inclusion and involvement of Indigenous people. However, for a range of valid reasons, including ethical and privacy‐related concerns, Indigenous people globally have consistently resisted genetic research (Taitingfong et al. 2020). Initiatives aimed at improving the cultural appropriateness, availability, access, and adaptability of genetic technologies and genomic research remain limited (Garrison, Hudson, et al. 2019). Concerns from Indigenous peoples surrounding a lack of co‐design or engagement (Behring et al. 2019), inadequate informed consent (Boyer et al. 2011), a genuine fear of exploitation (Boyer et al. 2011), and harmful, negative representation from genetic research have been highlighted (Garrison, Hudson, et al. 2019). Several reasonable solutions embedded within cultural context have been detailed previously in the literature, including co‐designed, co‐led, and research‐informed ethical frameworks (Caron et al. 2020). Studies have shown the need for explicit discussions with Indigenous communities that foster community‐engaged research to build genetic research capacity and genomic knowledge based on Indigenous partnerships (Hiratsuka et al. 2020). Researchers must continue to tackle the ethical, privacy‐related, and technical issues that impede the conduct of genomic analysis in order to provide the foundations of precision medicine within vulnerable and/or distinct population groups, thereby ensuring inclusivity in future clinical care opportunities (Pratt et al. n.d.). To begin to understand the possible contributions of precision medicine approaches to Indigenous populations, it is necessary to review extant research in this space and determine the scope for pharmacogenomics and genomics research.

## 2 Aims

This systematic review aimed to synthesize global empirical evidence involving Indigenous populations for genomics research with a particular focus on pharmacogenomics. The overarching intent of this review is to provide insight regarding how research has been conducted, the role of Indigenous participants in such research, and the benefits or outcomes achieved for Indigenous participants and/or communities.

## 3 Methodology

The protocol for this systematic review was registered on the Prospero database (CRD42021257226) and was conducted and reported using the Preferred Reporting Items for Systematic Reviews and Meta‐Analyses (PRISMA) protocol for systematic reviews (Moher et al. 2009).

### 3.1 Search Strategy

A systematic search was conducted using the following databases: PubMed, Medline, Embase, Cochrane, Scopus, CINAHL, and Web of Science, with search strategies being adjusted according to the requirements for each database. These searches were undertaken using keywords that related to the following themes: (i) Indigenous populations, (ii) pharmacogenomics, and (iii) precision medicine, with related terms and Medical Subject Headings. A pilot search was initially conducted, using a much longer list of both general and specific search terms to identify different Indigenous populations and possible key terms for inclusion. A test search of each term was performed by removing the term, re‐running the search, and comparing the results. If the removal of a given term did not alter the number of records returned, then the removed term was considered redundant and was not included in the final search string.

Results were restricted to peer‐reviewed articles in the English language, and articles published between January 2010 and July 2022. The initial search was conducted in June 2021 and updated in July 2022. Letters, editorials, or opinion pieces were not included as part of this systematic review (See Appendix 1 for full search strategy and MeSH terms).

### 3.2 Inclusion Criteria

To be eligible for inclusion, studies needed to meet several criteria as described in Table 1. Any study design (i.e., cross‐sectional, longitudinal, survey, experimental, program evaluation, qualitative, or mixed methods) that intentionally included commentary or analysis of the outcomes of empirical Indigenous genomics was eligible for inclusion. Empirical research included any qualitative, quantitative, or mixed methods research studies. Qualitative studies included interviews, open‐ended surveys, participants' observations, or focus groups. Mixed method studies were only considered if data from the quantitative or qualitative components could be clearly extracted. Studies were only included if findings were analyzed, reported, or discussed separately. No literature or systematic reviews were included in the results of this systematic review. Commentaries, perspectives, letters, reviews, editorials or opinion pieces, or grey literature were excluded.

**TABLE 1:** Inclusion and exclusion criteria.

Inclusion criteria	Exclusion criteria
Original peer‐reviewed empirical research, defined as any qualitative^a^, quantitative, or mixed methods research studies	Abstract or full‐text articles were unavailable, or methodology was not reported or incomplete
Eligible study designs included cross‐sectional, longitudinal, survey, experimental, program evaluation, qualitative, or mixed methods studies	Study did not report an eligible study design, for example, case series, case studies, conference proceedings/posters or abstracts, editorials, commentaries, perspectives, book chapters, dissertations, meta‐analyses and other systematic reviews, or grey literature
Study participants were identified as Indigenous, native, First Nations populations	Study did not involve any Indigenous populations
Study was conducted within the last 12 years and published in the English language	Study was not conducted within the past 12 years or not in the English language
Study reported pharmacogenomics and/or genomics research	Study did not report pharmacogenomics and/or genomics research

^a^ Qualitative studies could include interviews, open‐ended surveys, participants' observations, focus groups, and case studies. Mixed method studies were only considered if data from the quantitative or qualitative components could be clearly extracted.

### 3.3 Screening

Abstracts and titles were screened independently by two reviewers (BN and RV) to ensure the studies met the inclusion criteria. Any discrepancies regarding study eligibility were resolved through discussion with a third reviewer (KMR). All three reviewers (BN, RV, and KMR) discussed and agreed on all studies to be included in this review. Of the remaining studies, full‐text articles were then screened and analyzed for relevance and eligibility by two reviewers (BN and RV).

### 3.4 Data Extraction and Analysis

Data extraction and content analyses for both quantitative and qualitative empirical studies were conducted independently by two reviewers (BN and RV). Data extracted included an overview of study characteristics describing the study population, methods, and key outcomes (Table 2). Two reviewers (BN and KMR) independently assessed the quality of studies using questions adapted from published criteria on the quality assessment of interview, focus group, and survey studies using the mixed methods appraisal tool (MMAT) (Tong, Sainsbury, and Craig 2007). This tool has been specifically designed for systematic appraisal efforts for systematic mixed studies reviews that include qualitative, quantitative, and mixed methods studies. Scoring was based on 12 criteria distributed across the following domains: (i) description of aims and objectives, (ii) description of methods, (iii) participant selection, (iv) data collection, (v) data analysis, (vi) reporting, and (vii) engagement. Based on these criteria, studies were identified as being of good or poor quality (Table 3).

**TABLE 2:** Study characteristics.

	Reference	Design	Location and Sample size	Population	Aims	Methods	Outcomes
Quantitative Studies	Fohner et al. (2013)	Quantitative cohort study	Native America	Individuals (*n* = 187) from the Confederated Salish and Kootenai Tribes (American Indian and Alaskan Native populations)	To identify genetic variations in the P450 (CYP) genes (namely CYP2D6, CRY2A5, CYP3A, and CYP2C9), which are primarily responsible for Phase 1 drug metabolism, in a population of Native Americans	Genomic sequencing	Results highlight the importance of pharmacogenomic research in AI/AN populations. Outcomes could help optimize drug therapy for this population. Number of variants: CYP2D6: 26 CYP3A4: 15 CYP3A5: 10 CYP2C9: 41
	2 Cox et al. (2014)	Quantitative cohort study	Australia	Geographically isolated community of Indigenous Australians (*n* = 119)	To identify the presence of genetic variations in cytokine genes between Indigenous Australians and Caucasians and to determine the frequency of certain cytokine gene polymorphisms between Indigenous Australian and Caucasian controls	Analyzing frequency of genotypes for cytokines associated with inflammation	Genotypes associated with inflammation were higher in Indigenous Australians than in Caucasians
	3 Fohner et al. (2015)	Quantitative cohort study	Alaska and Anchorage	188 American Indian and American Native people and 94 Yup'ik people	To investigate variation in CYP2C9, VKORC1, CYP4F2, CYP4F11, and GGCX, which encode enzymes important for the activity of warfarin and synthesis of vitamin K‐dependent blood clotting factors	Resequencing of genes in 188 AI/AN people, 97 Yup'ik people and genotyping for specific SNPS in larger cohorts of each study population	This study predicts a lower average warfarin dose requirement in AI/AN populations in Alaska than that seen in non‐AI/AN populations of the United States, a finding consistent with clinical experience in Alaska
	4 Tanner et al. (2017)	Quantitative cohort study	Dakota and Arizona, USA	American Indians (*n* = 636) from two diff tribal populations in Dakota (Northern Plain AI) and Arizona (Southwest AI)	To compare CYP2A6 genetic variation and CYP2A6 enzyme activity (representative of the rate of nicotine metabolism) between the two tribal populations, as these have previously been associated with differences in smoking, quitting, and lung cancer risk	Participants were genotyped for CYP2A6 genetic variants *1B, *2, *4, *7, *9, *12, *17, and *35. Plasma and/or saliva samples were used to measure the nicotine metabolite ratio (NMR).	The overall frequency of genetically reduced nicotine metabolizers, those with CYP2A6 decrease‐ or loss‐of‐function alleles, was lower in the NP compared to the SW (*p* = 0.0006). CYP2A6 genotype was associated with NMR in both tribal groups (NP *p* < 0.001, SW *p* = 0.04). Notably, the rate of nicotine metabolism was higher in NP compared to SW smokers
	5 O'Connell et al. (2018)	Quantitative cohort study	South Africa	Black African (*n* = 77) and South African Coloured (*n* = 48) all female. Individuals of Black African origin, including Zulu (*n* = 101), Tswana (*n* = 45), Sotho (*n* = 42), and Xhosa (*n* = 31)	To characterize the genetic variations in genes involved in metabolizing and transporting antiretroviral drugs in a population of South African individuals	Genetic variations in genes involved in antiretroviral drug response	Fifty‐three variants had significant differences in allele and genotype frequencies when comparing SAC and BA groups. Thirteen of these have strong clinical annotations, affecting efavirenz and tenofovir pharmacokinetics. The observed differences between Indigenous population groups, and between these groups and global populations, demonstrate that data generated from specific African populations cannot be used to infer genetic diversity within other populations on the continent

	6 Naranjo et al. (2018)	Quantitative cohort study	Latin American countries. Regions of Mexico in North America, Nicaragua and Costa Rica from Central America, Cuba, and Peru, Ecuador, Colombia, Brazil, Argentina, Uruguay, Portugal, and Spain	Native Americans (*n* = 1395) locally identified to be Indigenous and living in areas with large indigenous populations	Characterizing the differences in the interpopulation variation in the CYP2D6, CYP2C19, and CYP2C9 gene in Latin American and Native American populations	To characterize interindividual and between population variations in CYP2D6, CYP2C9, and CYP2C19 drug metabolizing enzyme genotypes	Native Americans showed differences from each ethnic group in at least two alleles of CYP2D6, CYP2C9, and CYP2C19. Native Americans had higher frequencies of wild‐type alleles for all genes, and lower frequency of CYP2D6*41, CYP2C9*2, and CYP2C19*17 (*p* < 0.05). Native Americans also showed less CYP2C19 gUMs than the rest of the population sample. The interethnic differences described supports the need for population‐specific personalized and precision medicine programs for Native Americans
	7 Nagar et al. (2019)	Quantitative cohort study	Colombia	Individuals belonging to Antioquia and Chocó groups	To determine the genetic characteristics of individuals from neighboring populations in Colombia, with reference to admixture from Native Americans	A survey of the global distribution of pharmacogenomic variants followed by a more focused study of pharmacogenomic allele frequency differences between the two Colombian Antioquia and Chocó populations	This study found pharmacogenomic variants to have both unusually high minor allele frequencies and high levels of population differentiation. A number of these pharmacogenomic variants also show anomalous effect allele frequencies within and between the two Colombian populations, and these differences were found to be associated with their distinct genetic ancestry profiles
	8 Begnaud et al. (2020)	Quantitative case–control	The United States of America	Individuals self‐identifying as Native American with lung cancer (*n* = 164, 46.7% female). Mean age of 65.1 (SD 9.6) years	Determining the frequency of testing of genetic mutations in American Indians with adenocarcinoma and incidence of targeted gene therapy to treat lung cancer. Additional aims were to characterize the histopathology of lung cancer and common mutations causing lung cancer	Lung cancer mutational testing for somatic cancer mutations for American Indian/American Native populations	There was no significant difference in mutation testing in AI compared to non‐AI controls at large health care systems in Minnesota. These data indicate that other factors are likely contributing to the higher mortality in this group
9 de Carvalho et al. (2020)	Quantitative cohort study	Brazilian Amazonia	Two hundred and three healthy individuals from three Amerindian groups resident in the Brazilian Amazon region. Twenty‐seven were diagnosed with acute lymphoblastic leukemia	To investigate 27 molecular markers related to the treatment of acute lymphoblastic leukemia in Amerindians from Brazilian Amazonia and compare the frequencies with those recorded previously on five continents, that are available in the 1000 genomes database	Genotyping of the polymorphisms	Significant differences were found in the frequencies of most markers between the Amerindian populations and those of other regions around the world. Nine of the 12 markers investigated in the present study vary significantly in the Amerindian population in comparison with all five continental populations. The genetic profile of the Amerindian population is most like those of the American (AMR) and South Asian (SAS) populations

	10 Zhernakova et al. (2020)	Quantitative cohort study	Russia	Healthy, adult, ethnic Russians (*n* = 264)	To understand genome‐wide variations of healthy adults from Russia	Analysis of whole‐genome sequence data from 264 healthy adult ethnic Russians	This study presents analyses of the whole genome sequences of 60 newly sequenced individuals from three populations: Pskov region (western Russia), Novgorod region (western Russia), and Yakutia (eastern Siberia), and compared these to 204 individuals from 52 populations including both Russians and other ethnic groups
	11 Xhakaza et al. (2020)	Quantitative cohort study	South Africa	Indigenous Nguni population in South Africa	To investigate the association between genetic variants and responsiveness to medication for diabetic patients from the Indigenous Nguni population in South Africa	Genotyping 19 biomarkers for 140 Type 2 diabetic outpatients	The CT genotype of the rs12752688 polymorphism was significantly associated with increased response to metformin therapy after correction (OR = 0.33, 95% CI [0.16–0.68], *p*‐value = 0.006). An association was also found between the GA genotype of SLC47A2 rs12943590 and a decreased response to metformin therapy after correction (OR = 2.29, 95% CI [1.01–5.21], *p*‐value = 0.01)
	12 Ioannidis et al. (2020)	Quantitative Cohort study	Islands of Polynesia	Eight hundred and seven individuals from 17 island populations and 15 Pacific coast Native American groups	To analyze genome‐wide variation in individuals from islands spanning Polynesia for signs of Native American admixture	Analysis of genome‐wide variation in individuals from islands across Polynesia for signs of Native American admixture	This study presents conclusive evidence for prehistoric contact of Polynesians with Native Americans (ca. 1200 CE) contemporaneous with the settlement of remote Oceania
	13 Fernandes et al. (2022)	Quantitative cohort study	Brazil	Native Americans, and Kaingang and Guarani adults from Indigenous Reservation areas in Brazil (*n* = 694)	To identify single‐nucleotide variants with high predictive value as human leukocyte antigens proxies of the Americas	Genotype sequencing	This is the first study of the predictive performance of previously identified SNP tags for HLA‐A*31:01 in indigenous populations of the Americas, represented in the HGDP project and recruited from three reservations areas in Brazil. The diversity of cohorts is reflected in the range of frequencies of HLA‐A*31:01 (0.02–0.65), rs1061235 (0.03–0.13), and rs17179220 (0.12–0.66), as well as in the predictive performance of these SNVs as HLA‐A*31:01 proxies
14 Jaya Shankar et al. (2022)	Quantitative cohort study	Australia	Indigenous Australians (*n* = 187) from the Tiwi Islands	To understand the frequencies of pharmacologically relevant genetic variants within Indigenous populations of Australia	Analysis of whole‐genome sequence data from 187 individuals from the Tiwi Islands	This study identified 22 translationally relevant pharmacogenomic variants and 18 clinically actionable guidelines with implications for drug dosing and treatment of conditions including heart disease, diabetes, and cancer. It specifically observed increased poor and intermediate metabolizer phenotypes in the CYP2C9 (PM:19%, IM:44%) and CYP2C19 (PM:18%, IM:44%) genes

15 Moreira et al. (2022)	Quantitative cohort study	America	Nine hundred and fifty‐one healthy individuals from eight Native Latin American populations	To study the ancestry and PDE4B diversity in Native American populations along the American continent	Genotype sequencing	Findings from this study inform the discussion on the pertinence of an extra‐phase of chemotherapy in Native American populations and exemplifies how knowledge generated in US‐Hispanics is relevant for their even more neglected and vulnerable Native American ancestors along the American continent
Qualitative Studies	16 Shaw et al. (2013)	Qualitative focus groups	Alaska	Native Alaskans (*n* = 32, 38% male) attending an Alaska Native Primary Care Center. 47% aged 18–39 years, with 53% aged ≥ 40 years	To understand the opinions of Native American people, particularly in terms of their concerns and priorities, in doing pharmacogenetics in healthcare systems providing care for Native Americans in Alaska	Four focus groups	Pharmacogenetics was generally seen as benefiting and harming individuals, communities, and health systems, depending on methods and oversight
	17 Sahota (2014)	Qualitative interviews	Native American communities in SW USA	Native American community members (*n* = 53)	To examine the relationship (past and present) between the tribe and biomedical/genetics	Purposeful recruited past research participants and nonparticipants, as well as people with and without diabetes, and tribal members	This study provides insight into the understanding of the cultural logic related to specimen disposition. The process of informed consent and study procedures can be adapted to be culturally respectful of Native American communities
	18 Taualii et al. (2014)	Qualitative focus groups	Hawai'i	Native Hawaiians (*n* = 92)	To explore Native Hawaiian perceptions of and expectations for biobanking	Ten focus groups	Findings suggest that biobanking should be guided by six principles that comprise “G.R.E.A.T. Research” (Governance, Re‐consent, Education, Accountability, Transparency, Research priorities)
	19 Hudson et al. (2016) 20 Beaton et al. (2017)	Qualitative interviews and workshops	New Zealand	Maori and non‐Maori—panel members of an international advisory panel constituting members from Indigenous communities in Australia, Hawaii, Canada, US and NZ	To explore Maori views on biobanking and genomic research, and to identify ways to address Maori concerns over the collection and use of human tissue	Semi‐structured interviews with seven advisory panel members via telephone or skype	Maori and non‐Maori key informants highlighted the need to enhance levels
	21 Beans et al. (2018)	Qualitative mixed methods, survey with discussion groups	The United States of America	American Native and American Indian (*n* = 31) participating in a pharmacogenomics program	To characterize the preferences of Native Americans in disseminating/translating pharmacogenomics research	Descriptive statistics was used to analyze survey results. Thematic analysis was used to analyze discussion group data.	Participants were optimistic about pharmacogenetics research and its potential benefits. Participants requested their genetic information be provided to their health care providers and that they be kept up to date with this information

	22 Morgan et al. (2019)	Qualitative Focus groups	Canada	Individuals self‐identifying as Indigenous Canadians (*n* = 30); 30 participated in first focus group with 20 in the second group	To understand the views of Indigenous Canadians about their lack of representation in genomics research	Audio‐recording of focus groups including 30 First Nations, Metis, and Inuit individuals living in Greater Vancouver. After watching an introductory video explaining genomic testing, participants discussed issues surrounding collecting Indigenous genomic data, its control, and usage	Twenty participants who provided feedback concurred with the thematic interpretation: Systemic racism interlaced most conversations, particularly within the theme of trust. Some participants thought a separate, Indigenous‐controlled database was essential; others recognized advantages of international databases. The theme of implementation included creative ideas to collect Indigenous genomes, but prior approval from Indigenous leaders was emphasized
	23 Dirks et al. (2019)	Qualitative thematic analysis	Alaska	Rural Alaska Native community (*n* = 28)	To examine a rural Alaska Native community's viewpoint about biospecimen collection and storage; interest and recall in reporting family health history; and interest and engagement in biospecimen collection for conducting a genetic test for cancer	Four focus groups	Study participants shared interest in engaging in genetic cancer research and suggested ways to improve community engagement in research. These included transparency and continuous communication with researchers at all stages of the research, clear communication about the intent of the research, and that research and results take into consideration the community's needs
	24 Garrison, Barton, et al. (2019)	Qualitative semi‐structured interviews	Native American	American Indian/Alaska Native/Native Hawaiian tribal leaders, clinicians, researchers, policy makers, and tribal research review board members (*n* = 42)	To gauge perspectives on ethical issues related to genetics and genomic data sharing in American Indian/Alaska Native/Native Hawaiian communities	Sixty‐min semi‐structured interviews were conducted in‐person or via telephone and audio‐recorded with verbal consent	Appropriate data oversight and management of genetic data, obligations and control over sharing and accessing genomic data were key themes identified in this study
25 Ridgeway et al. (2019)	Qualitative focus groups	Florida, USA	African American, Hispanic/Latino, and Native American; 53 women and 15 men participated	To understand genetic research perceptions among members from underrepresented communities by studying discourse and language use in focus group discussions	Nine focus groups	Discourse analysis highlighted how conceptualization of science and family—rooted in historical experiences—can influence views on genetic research risks and benefits to self and others. Content analysis highlighted differences between the language use of focus group moderators, who spoke about scientific discovery and research oversight, and that of participants, whose talk highlighted ancestral bloodline, personal risk, and ethical concerns

	26 Hiratsuka, Brown, and Dillard (2012) 27 Hiratsuka et al. (2012)	Qualitative focus groups	Alaska	Alaskan community members (*n* = 82) and tribal leaders (*n* = 81)	To explore the views of biobanking research among Alaskan Native community members and leaders across Alaska using a community‐based participatory research approach with tribal and federal partners	Twenty‐nine focus groups were conducted in 14 locations	This study offers considerations for researchers partnering with Alaskan Native communities when planning research and public health surveillance projects. Our findings offer a guide for researchers and communities when planning and implementing research with biological specimens
	28 Hiratsuka et al. (2020)	Qualitative interviews	The United States of America	Native and non‐Native Americans (*n* = 11) involved in a research partnership aged 32–73 years. Mix between academic and non‐research backgrounds	To describe and assess a longstanding, complex research partnership between Indigenous and academic pharmacogenetic research partners, with attention to co‐learning and capacity building lessons learned	Descriptive thematic analysis of 11 semi‐structured interviews with interdisciplinary research partners situated at Indigenous and academic settings	Lessons learned included the need for explicit negotiation around mentoring expectations, and discussion on advisory and staff roles. Partners need to be aware not only of the structures, policies, and hierarchies within each partner institution, but also the tacit value commitments and understandings entailed in their different missions
	29 Rosas et al. (2020)	Qualitative mixed methods	The United States of America	One hundred community members (American Indian [*n* = 17], African American [*n* = 13], Chinese [*n* = 17], Latino [*n* = 27], and Vietnamese [*n* = 26]) and 14 physicians completed the survey and participated in the focus groups	To understand the views of ethnic minorities, including Native Americans, toward precision health research and practice among American Indian, African American, Latino, Chinese, and Vietnamese groups and physicians that serve these communities	A survey assessed demographics and opinions of precision health, genetic testing, and precision health research. Focus groups (*n* = 12) with each racial/ethnic minority group and physicians further explored attitudes about these topics	Familiarity with precision health was low among community members and high among physicians. Most groups were enthusiastic about the approach, especially if it considered influences on health in addition to genes (e.g., environmental, behavioral, social factors). Significant concerns were expressed by African American and American Indian participants about precision health practice and research based on past abuses in biomedical research
30 Nasir, Vinayagam, and Rae (2022)	Qualitative interviews	Australia	*N* = 48 Indigenous Australians with a diagnosed common mental disorder and comorbid chronic disease	To understand the needs and concerns regarding genomics research to appropriately tailor and help guide future personalized pharmacogenomics research approaches that lead to better treatment options for Indigenous chronic disease patients	Using community‐based participatory research principles, this study purposefully interviewed participants with a diagnosed common mental disorder and a comorbid chronic disease condition	Five emerging themes were identified, primarily focusing on the following: (Bayer and Galea 2015) The perceptions and understanding of genetics research; (Collins and Varmus 2015) culturally appropriate conduct of genetics research; (Landry et al. 2018) the role of indigenous‐led genetics research; (Bonham, Green, and Pérez‐Stable 2018) prospects of genetics research; and (Perera 2019) the importance of genetics research for patients with mental and physical health comorbidities

**TABLE 3:** Quality assessment using the mixed methods appraisal tool.

Studies	Criteria from the mixed methods appraisal tool																								
	1.1	1.2	1.3	1.4	1.5	2.1	2.2	2.3	2.4	2.5	3.1	3.2	3.3	3.4	3.5	4.1	4.2	4.3	4.4	4.5	5.1	5.2	5.3	5.4	5.5
Fohner et al. (2013)											Y	Y	Y	Y	Y										
2 Shaw et al. (2013)	Y	Y	Y	Y	Y																				
3 Cox et al. (2014)											Y	Y	Y	Y	Y										
4 Sahota (2014)	Y	Y	Y	Y	Y											Y	Y	Y	Y	CT	N	Y	Y	Y	Y
5 Taualii et al. (2014)	Y	Y	Y	Y	Y																				
6 Fohner et al. (2015)											Y	Y	Y	Y	Y										
7 Hudson et al. (2016) 8 Beaton et al. (2017)	Y	Y	Y	Y	Y																				
	Y	CT	CT	CT	CT																				
9 Tanner et al. (2017)											Y	Y	Y	Y	Y										
10 Beans et al. (2018)	CT	Y	CT	CT	CT																				
11 O'Connell et al. (2018)											Y	Y	Y	Y	Y										
12 Naranjo et al. (2018)											Y	Y	Y	Y	Y										
13 Morgan et al. (2019)	Y	Y	Y	Y	Y																				
14 Nagar et al. (2019)											Y	Y	Y	Y	Y										
15 Dirks et al. (2019)	Y	Y	Y	Y	Y																				
16 Garrison, Barton, et al. (2019)	Y	Y	Y	Y	Y																				
17 Ridgeway et al. (2019)	Y	Y	Y	Y	Y																				
18 Hiratsuka et al. (2012, 2012) 19 Hiratsuka et al. (2012, 2012) 20 Hiratsuka et al. (2020)	Y	Y	Y	CT	Y																				
	Y	Y	Y	CT	Y																				
	Y	Y	Y	Y	Y																				
21 Rosas et al. (2020)	Y	Y	Y	Y	Y											Y	N	Y	N	N	Y	Y	Y	U	Y
22 Begnaud et al. (2020)											Y	Y	Y	Y	Y										
23 de Carvalho et al. (2020)											Y	Y	Y	Y	Y										
24 Zhernakova et al. (2020)											Y	Y	Y	Y	Y										
25 Xhakaza et al. (2020)											Y	Y	Y	CT	Y										
26 Ioannidis et al. (2020)											Y	Y	Y	Y	Y										
27 Nasir, Vinayagam, and Rae (2022)	Y	Y	Y	Y	Y																				
28 Fernandes et al. (2022)											Y	Y	N	Y	CT										
29 Shankar et al. (2022)											Y	Y	Y	Y	Y										
30 Moreira et al. (2022)											Y	Y	Y	Y	CT										
31 Farinango et al. (2022)											Y	Y	Y	Y	Y										

## 4 Results

**FIGURE 1:**
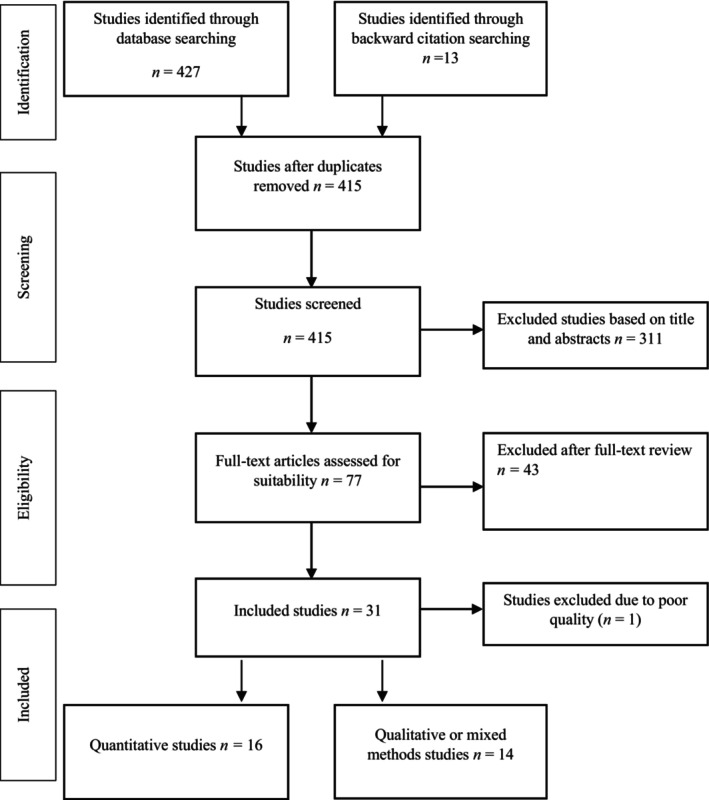
PRISMA chart of study search and selection process.

Initial searches retrieved 427 articles (Figure 1). After removing duplicates, 415 articles remained. Manual screening of titles and abstracts resulted in the selection of 77 articles that underwent full‐text screening and were reviewed for their eligibility and relevance to this systematic review. Full‐text screening resulted in a final 30 studies that were included in the final review (Table 4). Of these, 16 were quantitative studies (Jaya Shankar et al. 2022; Fohner et al. 2013, 2015; Cox et al. 2014; Tanner et al. 2017; Nagar et al. 2019; Begnaud et al. 2020; de Carvalho et al. 2020; Zhernakova et al. 2020; Xhakaza et al. 2020; Ioannidis et al. 2020; Fernandes et al. 2022; Moreira et al. 2022; Farinango et al. 2022; O'Connell et al. 2018; Naranjo et al. 2018) while 14 were identified as qualitative (Hiratsuka et al. 2012, 2020; Sahota 2014; Taualii et al. 2014; Hudson et al. 2016; Morgan et al. 2019; Dirks et al. 2019; Garrison, Barton, et al. 2019; Ridgeway et al. 2019; Hiratsuka, Brown, and Dillard 2012; Shaw et al. 2013) or mixed methods studies (Ridgeway et al. 2019; Nasir, Vinayagam, and Rae 2022; Beaton et al. 2017).

**TABLE 4:** Indigenous community engagement and participation in genomics research using the social‐ecological framework.

Socioecological model level	Theme	Promoters or enablers	Challenges or barriers
Intrapersonal	*Existing perceptions toward genomics research*	Willingness among non‐Indigenous and Indigenous academics to push back against university policies that do not align with culturally appropriate research (Hiratsuka, Brown, and Dillard 2012) Indigenous communities have positive perceptions on potential of genetics research to provide more efficacious ways of diagnosing and treating chronic diseases (Hudson et al. 2016; Morgan et al. 2019; Dirks et al. 2019; Shaw et al. 2013; Nasir, Vinayagam, and Rae 2022), and universally broader benefits for community (Morgan et al. 2019), particularly weighed against spiritual loss of tissue (Sahota 2014) Research participation embodying collectivist Indigenous attitudes on helping community (Taualii et al. 2014; Hudson et al. 2016; Ridgeway et al. 2019; Hiratsuka, Brown, and Dillard 2012; Nasir, Vinayagam, and Rae 2022) Increased knowledge of genetics research and what it involves for Indigenous community participants (Hudson et al. 2016)	Low perceived utility and benefits of genomics research (Morgan et al. 2019; Shaw et al. 2013) compared with socioeconomic and health programs of greater priority (Hudson et al. 2016; Morgan et al. 2019) Mistrust toward medical/genetics from past negative experiences with healthcare systems (Hiratsuka, Brown, and Dillard 2012; Rosas et al. 2020) and past ethical violations against Indigenous communities in research activities (Taualii et al. 2014; Hudson et al. 2016; Morgan et al. 2019; Rosas et al. 2020) Research findings may negatively impact Indigenous beliefs, knowledge, and ways of knowing (Ridgeway et al. 2019; Hiratsuka, Brown, and Dillard 2012)
	*Individual ownership of research*	Access to clinical based results for use in medical history and records (Morgan et al. 2019; Dirks et al. 2019) Personal growth from participatory research paradigms (Hiratsuka et al. 2020; Shaw et al. 2013)	Fears of potentially mandating involvement in genetics research (Shaw et al. 2013) Conducting genetic research only with Indigenous populations increases the potential of treating Indigenous people as different from other populations (Shaw et al. 2013; Nasir, Vinayagam, and Rae 2022)
Interpersonal	*Accountability of researchers and institutes and safeguards against Indigenous exploitation*	Awareness and increasing understanding of Indigenous sovereignty and colonial trauma among academic partners (Hiratsuka et al. 2020) Indigenous communities as gatekeepers of researchers to ensure community priorities are upheld/addressed (Beaton et al. 2017) Creating researcher contracts as safeguards against unethical behaviors (Beaton et al. 2017) Transparency when discussing topics potentially used for stigmatization (e.g., alcohol use) (Hiratsuka et al. 2020) and acknowledgment of past research abuses against Indigenous communities	Apprehension toward and distrust of researcher's motives for pursuing genetics research (Hiratsuka et al. 2012) Fear of potential for stigmatizing results and misuse for discrimination (Sahota 2014; Hudson et al. 2016; Morgan et al. 2019; Hiratsuka, Brown, and Dillard 2012; Shaw et al. 2013; Nasir, Vinayagam, and Rae 2022) Lack to efforts to recognize and understand Indigenous culture, leadership and expertise (Hiratsuka, Brown, and Dillard 2012) Indigenous communities as gatekeepers of researchers involved in projects, whereby those with records of past infringements barred (Beaton et al. 2017)
	*Meaningful continuation of researchers' involvement post data completion*	Building *strong* relationships between Indigenous communities and academic researchers (Hiratsuka et al. 2020) and sustaining them on completion of data collection (Dirks et al. 2019) Confidentially informing individuals where clinically significant results identified and providing access to further support (e.g., genetic counselling) (Ridgeway et al. 2019; Beaton et al. 2017) Regular updates on research progress, aims and results (Taualii et al. 2014; Dirks et al. 2019; Beaton et al. 2017; Hiratsuka et al. 2012), including secondary uses of data (Beaton et al. 2017)	Research participation only beneficial for individuals in the community or researchers (Dirks et al. 2019) Research fatigue from oversaturation of research projects involving Indigenous communities (Morgan et al. 2019) Lack of follow‐up post sample collection, in clinical and research‐settings and subsequent lack of knowledge transfer to individuals and communities e (Dirks et al. 2019; Hiratsuka et al. 2012; Hiratsuka, Brown, and Dillard 2012)
Organizational	*Indigenous leadership and ownership of research*	Academic partners acting in an advisory role to provide technical advice and support on project tasks as needed to meet project timelines) (Hiratsuka et al. 2020) Indigenous leaders and group leading and guiding all aspects of research, from conceptualization to implementation (Hiratsuka et al. 2020; Hudson et al. 2016; Morgan et al. 2019; Dirks et al. 2019; Shaw et al. 2013; Nasir, Vinayagam, and Rae 2022; Beaton et al. 2017) Non‐Indigenous Researchers receiving training to develop better cultural competence (Hudson et al. 2016; Hiratsuka et al. 2012; Nasir, Vinayagam, and Rae 2022; Rosas et al. 2020) Indigenous academics leading research (Taualii et al. 2014; Morgan et al. 2019; Nasir, Vinayagam, and Rae 2022) Research focus and direction reflecting priorities of Indigenous communites (Taualii et al. 2014; Dirks et al. 2019; Nasir, Vinayagam, and Rae 2022; Beaton et al. 2017)	
	*Benefits for Indigenous communities*	Community development opportunities through involvement in genomics research (Hiratsuka et al. 2020; Hudson et al. 2016; Shaw et al. 2013) Hiring Indigenous community members in the project team (Morgan et al. 2019; Dirks et al. 2019) Building Indigenous communities' research capacity and self‐determination (e.g., offering scholarships and employment to Indigenous applicants in genomics field) (Hiratsuka et al. 2020; Shaw et al. 2013)	
	*Autonomy of Indigenous communities in research activities*	Informed consent and thorough understanding of all aspects of research, including data access, ownership and sharing (Hudson et al. 2016; Dirks et al. 2019; Garrison, Barton, et al. 2019; Beaton et al. 2017) (Hiratsuka et al. 2012; Hiratsuka, Brown, and Dillard 2012; Beaton et al. 2017) in accessible language (Hudson et al. 2016; Hiratsuka et al. 2012; Nasir, Vinayagam, and Rae 2022; Beaton et al. 2017) Precise and accurate wording to define and set the parameters of consent and data access (Beaton et al. 2017) Participation in research should be wholly voluntary (Dirks et al. 2019; Garrison, Barton, et al. 2019; Shaw et al. 2013) No coercion/pressure to data share where tribal members have misgivings about misuses (Garrison, Barton, et al. 2019) Option to withdraw consent at any time (Beaton et al. 2017) Transparency and openness underlying all research processes (Taualii et al. 2014; Hudson et al. 2016; Ridgeway et al. 2019; Beaton et al. 2017) Open and clear communication on research aims (Dirks et al. 2019) Robust policies on data sharing, confidentiality, and privacy (Morgan et al. 2019; Dirks et al. 2019; Garrison, Barton, et al. 2019; Hiratsuka, Brown, and Dillard 2012; Shaw et al. 2013; Rosas et al. 2020)	Past stigmatizing portrayal of Indigenous communities; mistrust and fears genetic research may be used to further marginalize Indigenous peoples (Hiratsuka, Brown, and Dillard 2012; Rosas et al. 2020)
	*Data management and accessibility*	Data management (Taualii et al. 2014; Hudson et al. 2016; Garrison, Barton, et al. 2019; Beaton et al. 2017) with data sharing contracts and policies developed in partnership with Indigenous community members (Garrison, Barton, et al. 2019; Beaton et al. 2017) Indigenous governance and ownership of data and control in its secondary use (Hudson et al. 2016; Morgan et al. 2019; Garrison, Barton, et al. 2019; Beaton et al. 2017) Indigenous choice of data management policies (Hudson et al. 2016; Morgan et al. 2019) Data sharing policies respecting community member's needs and preferences (Dirks et al. 2019) Development of Indigenous organizations for research oversight and goverance (Taualii et al. 2014) Acknowledgment and recognition of Indigenous communities when data is used (Beaton et al. 2017) Strict deterrents and policies against commodification and commercialization of data (Sahota 2014; Morgan et al. 2019; Ridgeway et al. 2019; Nasir, Vinayagam, and Rae 2022) Re‐consent process for every instance of data use (Taualii et al. 2014; Hiratsuka et al. 2012; Hiratsuka, Brown, and Dillard 2012; Beaton et al. 2017)	Past misuse of data without consent of Indigenous communities leads to apprehension with data sharing. Federal grant policies infringing on Indigenous communities having data ownership (Garrison, Barton, et al. 2019) Low trust in institutions responsible for data management (e.g., federal agencies) (Garrison, Barton, et al. 2019; Rosas et al. 2020) Lack of robust safeguards around data sharing (Garrison, Barton, et al. 2019)
	*Research and consent processes reflecting priorities and beliefs of Indigenous communities*	Respecting and acknowledging Indigenous beliefs on sacredness of biological materials in research processes and methods (Hudson et al. 2016), including protocols to return or destroy biological samples to participants where requested (Sahota 2014; Morgan et al. 2019; Beaton et al. 2017) Culturally appropriate and accessible research and consent processes (Hudson et al. 2016; Hiratsuka et al. 2012; Shaw et al. 2013; Beaton et al. 2017), including sensitivity to age‐based needs (Shaw et al. 2013) University policy and grant agreements aligning with Indigenous beliefs (e.g., in terms of data sharing) (Hiratsuka et al. 2020) Grant and funding criteria requiring inclusion of and consultation with Indigenous communities (Beaton et al. 2017) Researchers acknowledging and accepting Indigenous expertise (Hiratsuka et al. 2020) Consulting and introducing research through trusted Indigenous community members (Hudson et al. 2016; Nasir, Vinayagam, and Rae 2022) Cultural awareness when discussing sensitive topics (e.g., family history and deceased individuals) (Nasir, Vinayagam, and Rae 2022) Awareness of gender roles in Indigenous communities, possibility participants may be more comfortable talking to researchers of same‐sex (Perera 2019)	Lack of autonomy in choosing to engage in genetic research Homogenization of research across different Indigenous communities with lack of effort to recognize diversity across different Indigenous communities (Morgan et al. 2019; Hiratsuka, Brown, and Dillard 2012; Nasir, Vinayagam, and Rae 2022)
Societal	*Health and socioeconomic equity*	Producing efficacious and economic benefits for Indigenous communities and their future generations (Taualii et al. 2014; Hudson et al. 2016; Morgan et al. 2019; Ridgeway et al. 2019; Hiratsuka, Brown, and Dillard 2012; Shaw et al. 2013; Nasir, Vinayagam, and Rae 2022; Beaton et al. 2017) Potential for cost savings from more effective disease treatments to be re‐invested to improve health care for Indigenous peoples (Shaw et al. 2013) Genetics research as an avenue for regaining knowledge of family history and medical predispositions lost through intergenerational abuses and separation from family (Dirks et al. 2019; Ridgeway et al. 2019; Nasir, Vinayagam, and Rae 2022)	Genetics research would consume or divert funding from higher healthcare priorities (Hudson et al. 2016; Hiratsuka, Brown, and Dillard 2012; Shaw et al. 2013) Genetics research not being universally beneficial to Indigenous community members (Dirks et al. 2019; Shaw et al. 2013) Focus of genetic research/results detracts attention from systemic and colonial causes of ill health (Hudson et al. 2016)
	*Recognition of and respect for Indigenous “ways of knowing, being and doing”* (Martin and Mirraboopa 2003)	Understanding and acceptance of Indigenous beliefs and identity (Hiratsuka et al. 2020) Trust and acquaintance building through informal gatherings (e.g., sharing meals) (Hiratsuka et al. 2020) Longer partnership/acquaintance required to address sensitive topics (i.e., having a solid foundation of trust) (Hiratsuka et al. 2020)	Researchers are easily accessible to community members Communicating in an unclear and inaccessible manner, taking into consideration English may be one of many different languages spoken (Hudson et al. 2016; Nasir, Vinayagam, and Rae 2022) Accessibility and convenience of getting blood samples (Morgan et al. 2019)

### 4.1 Quality Assessment and Risk of Bias Analyses

Quality assessment and risk of bias for all quantitative studies, qualitative and mixed methods studies selected were assessed using the MMAT (Table 3). The MMAT has shown to have high validity (Hong et al. 2019) and reliability (Souto et al. 2015), and is a useful tool for appraising literature studies (Hong, Gonzalez‐Reyes, and Pluye 2018). All studies but one were of good quality and with low or moderate risk of bias (*n* = 30).

### 4.2 Location and Populations

Most studies were conducted with Native Indigenous populations from the United States of America (*n* = 11) (Hiratsuka et al. 2020; Fohner et al. 2013, 2015; Tanner et al. 2017; Begnaud et al. 2020; Moreira et al. 2022; Dirks et al. 2019; Garrison, Barton, et al. 2019; Shaw et al. 2013; Souto et al. 2015; Hong, Gonzalez‐Reyes, and Pluye 2018) with one study from among these including Latin American countries (Naranjo et al. 2018). There were five studies conducted with Indigenous Alaskan communities (Fohner et al. 2015; Dirks et al. 2019; Hiratsuka et al. 2012; Hiratsuka, Brown, and Dillard 2012; Shaw et al. 2013), of which one specifically included Alaskan Anchorage populations (Fohner et al. 2015). There were three studies conducted with Indigenous Australians (Jaya Shankar et al. 2022; Cox et al. 2014; Nasir, Vinayagam, and Rae 2022), two with native Brazilians (de Carvalho et al. 2020; Fernandes et al. 2022), two with New Zealand Māori (Hudson et al. 2016; Beaton et al. 2017), and two with Indigenous South African populations (Xhakaza et al. 2020; O'Connell et al. 2018). Other studies involved Indigenous populations from Russia (Zhernakova et al. 2020), Hawai'i (Taualii et al. 2014), Canada (Morgan et al. 2019), Islands of Polynesia (Ioannidis et al. 2020), Ecuador (Farinango et al. 2022), and Colombia (Nagar et al. 2019).

### 4.3 Content Analysis

Of the quantitative, case–control, and cohort studies (*n* = 16, 53%), 11 (37%) specifically detailed obtaining approvals via ethical research committee and participant consent. (Jaya Shankar et al. 2022; Fohner et al. 2013, 2015; Cox et al. 2014; Tanner et al. 2017; de Carvalho et al. 2020; Zhernakova et al. 2020; Xhakaza et al. 2020; Fernandes et al. 2022; Farinango et al. 2022; O'Connell et al. 2018; Naranjo et al. 2018) Seeking approval from Indigenous review boards or tribal groups/leaders was only undertaken by four of the quantitative studies (13%) (Jaya Shankar et al. 2022; Fohner et al. 2013, 2015; Cox et al. 2014; Tanner et al. 2017). Studies undertaking qualitative or mixed method methodologies (*n* = 14, 47%) included participation of Indigenous people mostly through research interviews (Garrison, Hudson, et al. 2019; Sahota 2014; Hudson et al. 2016; Nasir, Vinayagam, and Rae 2022; Beaton et al. 2017) (*n* = 6, 20%) or focus groups (Hiratsuka et al. 2020; Taualii et al. 2014; Morgan et al. 2019; Dirks et al. 2019; Ridgeway et al. 2019; Hiratsuka et al. 2012; Hiratsuka, Brown, and Dillard 2012; Shaw et al. 2013; Rosas et al. 2020; Beans et al. 2018) (*n* = 9, 30%).

Genomics research involving Indigenous populations was divided into two distinct areas of research. Studies either aimed to explore genetic variations among Indigenous populations that were associated with disease (Cox et al. 2014; Begnaud et al. 2020; de Carvalho et al. 2020; Zhernakova et al. 2020; Ioannidis et al. 2020; Fernandes et al. 2022; Naranjo et al. 2018) (*n* = 7, 23%), were important markers for drug metabolism (Jaya Shankar et al. 2022; Fohner et al. 2013, 2015; Tanner et al. 2017; Nagar et al. 2019; Xhakaza et al. 2020; Moreira et al. 2022; Farinango et al. 2022; O'Connell et al. 2018) (*n* = 9, 30%), or aimed to understand the perspectives of Indigenous populations regarding the conduct of genomics research (Garrison, Hudson, et al. 2019; Hiratsuka et al. 2020; Taualii et al. 2014; Hudson et al. 2016; Morgan et al. 2019; Dirks et al. 2019; Ridgeway et al. 2019; Hiratsuka et al. 2012; Hiratsuka, Brown, and Dillard 2012; Shaw et al. 2013; Nasir, Vinayagam, and Rae 2022; Beaton et al. 2017; Rosas et al. 2020; Beans et al. 2018) (*n* = 14, 47%). A significant number of studies focused on investigating genetic variations that explored CYP gene variations (Jaya Shankar et al. 2022; Fohner et al. 2013, 2015; Tanner et al. 2017; Naranjo et al. 2018) (*n* = 5, 17%).

In this systematic review, we aimed to explore the Indigenous perspective on how research was conducted, the role of Indigenous participants in that research, and the benefits or outcomes achieved for Indigenous participants and/or communities through genomics research. Outcomes described below revolve around these three main domains.

### 4.4 The Indigenous Perspective on Genomics Research

Studies exploring the role of Indigenous participants in genomics research mostly focused on maintaining mutual research priorities and health needs (Garrison, Hudson, et al. 2019; Taualii et al. 2014; Hudson et al. 2016; Ridgeway et al. 2019; Shaw et al. 2013; Nasir, Vinayagam, and Rae 2022; Beaton et al. 2017) (*n* = 7, 23%) at the forefront of all genomics research being conducted within Indigenous communities. Participants also highlighted the importance of incorporating Indigenous governance (Hiratsuka et al. 2020; Taualii et al. 2014; Hudson et al. 2016; Morgan et al. 2019; Ridgeway et al. 2019; Nasir, Vinayagam, and Rae 2022) (*n* = 6, 20%) and several specifically focused on control of data access and sharing (Sahota 2014; Hudson et al. 2016; Garrison, Barton, et al. 2019; Nasir, Vinayagam, and Rae 2022; Beaton et al. 2017) (*n* = 5, 16%). Ensuring informed consent (Sahota 2014; Taualii et al. 2014; Hudson et al. 2016; Hiratsuka et al. 2012; Nasir, Vinayagam, and Rae 2022; Beaton et al. 2017) (*n* = 6, 20%) and transparency across all research activities being conducted (Taualii et al. 2014; Hudson et al. 2016; Dirks et al. 2019; Hiratsuka et al. 2012) were also significant factors highlighted by participants. Community engagement (Hudson et al. 2016; Dirks et al. 2019; Beaton et al. 2017), necessary participant education (Taualii et al. 2014; Beans et al. 2018), and continuous communication with participants (Hudson et al. 2016; Dirks et al. 2019; Beans et al. 2018) were additional important considerations highlighted by participants in the studies reviewed. Other significant aspects included the need to establish mutual partnership (Hiratsuka et al. 2020; Nasir, Vinayagam, and Rae 2022), equal participation (Hiratsuka et al. 2012; Beaton et al. 2017), maintain trust (Hiratsuka et al. 2020; Morgan et al. 2019; Beaton et al. 2017), accountability (Hudson et al. 2016; Hiratsuka, Brown, and Dillard 2012), and reciprocity (Morgan et al. 2019). Ensuring culturally respectful research procedures (Dirks et al. 2019; Nasir, Vinayagam, and Rae 2022; Beaton et al. 2017) and incorporating “cultural logic” (Sahota 2014) were also emphasized.

A genuine concern regarding future unknown use or data sharing capabilities (Hiratsuka, Brown, and Dillard 2012; Rosas et al. 2020) and culturally suitable specimen disposal (Sahota 2014; Hiratsuka et al. 2012; Beaton et al. 2017) were priorities that emerged from the Indigenous populations involved in these studies. Studies also highlighted the continued worry and fear of potential discrimination or stigmatization (Morgan et al. 2019; Hiratsuka, Brown, and Dillard 2012; Shaw et al. 2013; Rosas et al. 2020), distrust (Morgan et al. 2019; Beaton et al. 2017; Rosas et al. 2020; Nasir, Vinayagam, and Rae 2022), and the security or confidentiality of genetic information (Morgan et al. 2019; Hiratsuka, Brown, and Dillard 2012; Rosas et al. 2020). Cost and affordability were also raised as a concern for some participants (Shaw et al. 2013; Rosas et al. 2020).

### 4.5 The Role of Indigenous Participants in Genomics Research

No study specifically explored the role of participants in genomics research involving Indigenous people or communities. Similarly, no studies specifically described Indigenous participant involvement in data collection or data analysis. However, a small number involved Indigenous participants in data interpretation (Morgan et al. 2019; Hiratsuka, Brown, and Dillard 2012) and used methodologies which were established by the Indigenous participants or with communities themselves (Jaya Shankar et al. 2022; Hiratsuka, Brown, and Dillard 2012). A significant number of studies acknowledged community members or participants within the study (Jaya Shankar et al. 2022; Garrison, Hudson, et al. 2019; Fohner et al. 2013, 2015; Cox et al. 2014; Nagar et al. 2019; Xhakaza et al. 2020; Ioannidis et al. 2020; Sahota 2014; Hudson et al. 2016; Morgan et al. 2019; Dirks et al. 2019; Ridgeway et al. 2019; Nasir, Vinayagam, and Rae 2022; Beaton et al. 2017) (*n* = 15, 50%); however, no study included an Indigenous community member or participant specifically as a co‐author.

### 4.6 Benefits or Outcomes Achieved From Genomics Research in Indigenous Populations

All studies identified in this review explored benefits or outcomes from the conduct of genomics research with Indigenous populations. Studies focused on genetic data analysis that contributed to identifying unique genetic variations and pharmacogenetics research (Jaya Shankar et al. 2022; Fohner et al. 2013, 2015; Cox et al. 2014; Tanner et al. 2017; Nagar et al. 2019; Begnaud et al. 2020; de Carvalho et al. 2020; Zhernakova et al. 2020; Xhakaza et al. 2020; Ioannidis et al. 2020; Fernandes et al. 2022; Moreira et al. 2022; Farinango et al. 2022; O'Connell et al. 2018; Naranjo et al. 2018) or engaged in understanding perspectives related to potential benefits or outcomes of genomics research (Hiratsuka et al. 2020; Sahota 2014; Taualii et al. 2014; Hudson et al. 2016; Morgan et al. 2019; Dirks et al. 2019; Garrison, Barton, et al. 2019; Ridgeway et al. 2019; Hiratsuka et al. 2012; Hiratsuka, Brown, and Dillard 2012; Shaw et al. 2013; Nasir, Vinayagam, and Rae 2022; Beaton et al. 2017; Rosas et al. 2020; Beans et al. 2018). A focus of genomics research was to facilitate beneficial outcomes for the better optimization of drug therapy for Indigenous people (Fohner et al. 2013, 2015; Nagar et al. 2019; Xhakaza et al. 2020; Moreira et al. 2022; O'Connell et al. 2018; Shaw et al. 2013; Beans et al. 2018; Henderson et al. 2018) (*n* = 9, 30%). However, other studies also explored harmful outcomes and potential risks of genomics research that could result from the misuse of genomic information (Hiratsuka et al. 2020; Dirks et al. 2019; Ridgeway et al. 2019; Shaw et al. 2013; Rosas et al. 2020). Some articles explored perspectives on genomic biobanks (Hiratsuka, Brown, and Dillard 2012; Beaton et al. 2017) (*n* = 4, 13%) and how understandings of specimen disposal (Sahota 2014) and security of confidentiality of genetic information (Rosas et al. 2020) can have beneficial genomics research outcomes for Indigenous communities.

### 4.7 Indigenous Engagement and Participation in Genomics Research

Using a social‐ecological framework (Nutbeam, Harris, and Wise 2010) that considers levels based on the complex interplay between individual, relationship, community, and societal factors, we appraised Indigenous engagement and participation in genomics research (Table 4). Also known as the socio‐ecological model (SEM), this conceptual framework is used for understanding the multiple levels of interrelated factors influencing health and health behaviours (Nutbeam, Harris, and Wise 2010). The SEM levels are commonly defined as the individual, interpersonal, community, organizational, and policy levels. The naming of these levels varies slightly, but the fundamental idea of this model is that public health efforts need to use a combination of interventions at all levels and across society. (Nutbeam, Harris, and Wise 2010; Schölmerich and Kawachi 2016) This model can also be applied to evaluate what elements of precision medicine/genomics research work for whom, why, and how in an Indigenous context. Furthermore, the SEM approach holds great potential for complementing the life‐course perspective to reducing existing disparities in health outcomes from birth for Indigenous populations (Schölmerich and Kawachi 2016).

Key outcomes from the systematic review have identified that there are enablers and challenges across the SEM levels, with the following themes: perceptions and ownership of research (individual and collective); accountability and safeguards; meaningful partnerships between researchers and communities; Indigenous leadership and governance; capacity building and sharing with communities; autonomy and consent over research processes (e.g., data management), and health and socioeconomic inequities and recognition of Indigenous ways of being, doing, and knowing. Promoters and barriers for each of these identified themes are outlined in Table 4.

Some papers were particularly strong, with enablers that were important in acknowledging Indigenous protocols and the respectful conduct of research with Indigenous communities (Hudson et al. 2016; Morgan et al. 2019; Beaton et al. 2017). Key informants in these studies spoke about the need to protect Indigenous, specifically Māori interests through Māori control, which promoted concepts of power‐sharing over benefit‐sharing (Sahota 2014; Hudson et al. 2016). (Beans et al. 2018) Across studies, individuals valued clear and ongoing communication, particularly in the context of past experiences involving a lack of knowledge transfer, both across clinical and research‐based settings. (Dirks et al. 2019; Hiratsuka et al. 2012; Hiratsuka, Brown, and Dillard 2012) This was a barrier in these studies which particularly highlighted the subsequent inaccessibility of gained knowledge (Dirks et al. 2019; Hiratsuka et al. 2012; Hiratsuka, Brown, and Dillard 2012). These safeguards about data sharing and data management (Taualii et al. 2014; Hudson et al. 2016; Garrison, Barton, et al. 2019; Beaton et al. 2017) were also highlighted as an important inclusion, with robust policies regarding data confidentiality, privacy, and the promotion of informed consent as major considerations (Hudson et al. 2016; Morgan et al. 2019) and omissions in some studies (Garrison, Barton, et al. 2019; Rosas et al. 2020).

## 5 Discussion

This review aimed to explore and describe genomics research being conducted with Indigenous participants globally. The findings reported in this review have identified a relatively small number of studies, highlighting a growing gap in Indigenous genomics research. Quantitative research studies had a strong focus on the understanding of a variety of drug metabolism concerns including nicotine metabolism (Tanner et al. 2017), Phase 1 drug metabolism pathways (Fohner et al. 2013, 2015; Naranjo et al. 2018), response to metformin (Xhakaza et al. 2020), and antiretroviral metabolism (O'Connell et al. 2018). However, there were also several other studies that were interested in genetic comparisons between populations including Antiquo and Choco communities (Nagar et al. 2019), Russian communities (Zhernakova et al. 2020), and groups in Latin America (Moreira et al. 2022). Inflammation was the focus of one study that considered polymorphisms in cytokine genes (Cox et al. 2014) and two further studies focused on specific illnesses, including lung adenocarcinoma (Begnaud et al. 2020) and acute lymphoblastic leukemia (de Carvalho et al. 2020). Qualitative and mixed methods studies explored Indigenous perspectives regarding genomics research conducted with Indigenous participants. No study specifically indicated the involvement of Indigenous participants, communities, or researchers as part of the initial study design, data collection or analysis, although a few reported incorporating participant feedback on the final outcomes. Studies acknowledge Indigenous involvement; however, a specific description of being Indigenous‐led or authored is limited. Harnessing genomic advancement for Indigenous communities to improve health and well‐being is not only a priority, but an essential way forward to enable Indigenous‐focused medical and health research.

This review highlights that the considerations for those conducting genomics research with Indigenous communities are many and varied and can be assessed across different SEM levels. The study team considered objectives for research with Indigenous communities needed to provide evidence of understanding of the Indigenous perspectives, the role of Indigenous participants, and the benefits of the achieved outcomes. Table 4 highlights which studies helped or hindered efforts to achieve these objectives. This was similarly found in the literature, with overall findings pointing to increased Indigenous control of research processes with a commitment to inclusion, reciprocity, and increasing opportunities for research excellence (Ewen, Ryan, and Platania‐Phung 2019; Australian Institute of Aboriginal and Torres Strait Islander Studies 2012).

Ensuring Indigenous communities are included in the design, implementation, analysis, and outcomes of genomics research is an important aspect that needs to be given more attention. Guidelines to implement ethical research and best practices within research involving Indigenous participants and communities that value, prioritize, and empower Indigenous traditional knowledge and equal participation have been developed for various populations across the globe (National Health and Medical Research Council (Australia) 2018; Australian Institute of Aboriginal and Torres Strait Islander Studies 2012; Hudson et al. 2010). However, without the active participation and governance of Indigenous consumers during all stages of research, the potential to disempower and misinterpret research outcomes can arise (MacLean et al. 2015; Drawson, Toombs, and Mushquash 2017). This review also identified the ongoing concern regarding the potential for discrimination, and the lack of trust, reciprocity, and transparent partnerships or engagement that continues to exist despite efforts to alleviate historical wrongdoing. Indigenous academic involvement and authorship inclusion is also important; this review highlights the limited acknowledgment and the inability to properly know if the authors were Indigenous. Acknowledgment and recognition of Indigenous authorship may warrant further review and consideration within Indigenous research.

Developing new models of leadership and governance and enabling functional transparent, best‐practice standards and operating protocols are a requirement to establish effective community engagement, informed consent, management, and use of biological samples and data for the ethical conduct and management of Indigenous genomic research projects. Researchers must be responsive to these needs and ensure that they produce high‐impact research evidence from which health benefits can follow. Policymakers and practitioners who utilize genomics research evidence need to understand with confidence the outcomes of their actions to make informed decisions that have the potential to result in positive health impact (McCalman et al. 2016) for Indigenous people. Ultimately, little progress can be made in the field of Indigenous genomics without specific attention to and investment in ensuring Indigenous leadership and control and ownership over genomic research, with a commitment to prioritizing genomics healthcare to enable the well‐being and better health of Indigenous people.

Findings of a narrative review on capacity building of Indigenous health researchers reported the need for improvements in collaborative research between Indigenous and non‐Indigenous researchers and organizations. This review discussed the need for a shift from a deficit‐ to strength‐based education and research focus with respect to both participation and quality, Indigenous health researchers to lead or co‐lead projects due to their commitment in research that makes a meaningful contribution to community wellbeing, and highlighted the importance of strengthening the research capabilities of community members (Ewen, Ryan, and Platania‐Phung 2019). Research capacity building is imperative to achieve gains in workforce development, improving health systems, and undertaking research studies with Indigenous communities.

### 5.1 Strengths and Limitations

A strength of this review is the inclusion of quantitative and qualitative papers in data extraction efforts and the assessment of all studies to systematically examine Indigenous community involvement by using a socio‐ecological framework. This approach highlighted that only a small number of studies sought approval from Indigenous review boards or tribal groups/leaders, providing additional insights into areas of need when designing pharmocogenomic research efforts in partnership with Indigenous communities. While this review included studies spanning a broad range of global regions, with the limited involvement of Indigenous researchers and organizations, the contextualization and interpretation of results may be lacking. Notably, this work is not representative of all global Indigenous populations due to the diversity of these communities and the dearth of literature from some regions.

## Author Contributions

B.F.N. contributed to the study conceptualization and design, conducting the review, data extraction, analysis, and appraisal, interpretation of the data, drafting the manuscript and revising it critically, and obtained the final approval of the version to be published. R.V. contributed to the data extraction, analysis and appraisal, interpretation of the data, drafting the manuscript and revising it critically, and obtained the final approval of the version to be published. L.M. contributed to the writing of the qualitative analysis, editing of the qualitative data table, and made additions to the manuscript and gave a final approval of the version to be published. M.T. contributed to the study conceptualization and design and revising the manuscript critically and gave a final approval of the version to be published. S.H.N. contributed to the study design and revising the manuscript critically and gave a final approval of the version to be published. K.M.R. contributed to the study design, data extraction, analysis and appraisal, interpretation of the data, and revising the manuscript critically, and gave a final approval of the version to be published.

## Conflicts of Interest

The authors declare no conflicts of interest.

## Data Availability

The authors have nothing to report.

## Appendix 1 Search Strategy

### 1.1

A systematic search was conducted using the following databases: PubMed, Medline, Embase, Cochrane, Scopus, CINAHL, and Web of Science. The search strategies were adjusted according to the requirements for each database, using keywords that were related to the following themes: (i) Indigenous populations, (ii) pharmacogenomics and/or genomics, and (iii) precision medicine.

A pilot search was initially conducted, using a much longer list of both general and specific search terms to identify different Indigenous populations and possible key terms for inclusion. A test search of each term by removing the term, re‐running the search, and comparing the results. If the removal of a given term did not alter the number of records returned, then the removed term was considered redundant, and was not included in the final search string.

The search strategy was developed in MEDLINE using the Population, Intervention/Phenomena of Interest, Comparison, Outcome (PICO) framework as described below. Relevant keywords and medical subject heading (MeSH) terms were then adapted for each database.

**Table:**

Framework	Description
Population	Indigenous populations from across the world
Intervention	Pharmacogenomics and/or genomics empirical research
Control	N/A
Outcome	Reported on pharmacogenomics and/or genomics research that involves Indigenous populations

#### PubMed

1. (((((australia[mh] OR australia*[tiab]) AND (oceanic ancestry group[mh] OR aborigin*[tiab] OR indigenous[tw])) OR (torres strait* islander*[tiab])) AND medline[sb]) OR ((((au[ad] OR australia*[ad] OR australia*[tiab] OR northern territory[tiab] OR northern territory[ad] OR tasmania[tiab] OR tasmania[ad] OR new south wales[tiab] OR new south wales[ad] OR victoria[tiab] OR victoria[ad] OR queensland[tiab] OR queensland[ad]) AND (aborigin*[tiab] OR indigenous[tiab])) OR (torres strait* islander*[tiab])) NOT medline[sb])) OR (((("Indigenous Peoples"[Mesh]) OR "Indians, North American"[Mesh]) OR "Inuits"[Mesh]) OR (((((((("first nation*"[Title/Abstract]) OR ("indigenous population*"[Title/Abstract])) OR (tribe[Title/Abstract])) OR (tribal[Title/Abstract])) OR (Maori[Title/Abstract])) OR ("Native American"[Title/Abstract])) OR (inuit*[Title/Abstract])) OR (metis[Title/Abstract])))
2. ((("Precision Medicine"[Mesh]) OR "Pharmacogenetics"[Mesh]) OR "Pharmacogenomic Variants"[Mesh]) OR ((((((("precision medicine"[Title/Abstract]) OR ("personalised medicine"[Title/Abstract])) OR ("personalized medicine"[Title/Abstract])) OR (genetic*[Title/Abstract])) OR (genomic*[Title/Abstract])) OR (pharmacogenetic*[Title/Abstract])) OR (pharmacogenomic*[Title/Abstract]))
3. Search: ("Health"[Mesh]) OR (health[Title/Abstract])
4. (((((("australia"[MeSH Terms] OR "australia*"[Title/Abstract]) AND ("oceanic ancestry group"[MeSH Terms] OR "aborigin*"[Title/Abstract] OR "indigenous"[Text Word])) OR "torres strait islander*"[Title/Abstract]) AND "medline"[Filter]) OR (((("au"[Affiliation] OR "australia*"[Affiliation] OR "australia*"[Title/Abstract] OR "northern territory"[Title/Abstract] OR "northern territory"[Affiliation] OR "tasmania"[Title/Abstract] OR "tasmania"[Affiliation] OR "new south wales"[Title/Abstract] OR "new south wales"[Affiliation] OR "victoria"[Title/Abstract] OR "victoria"[Affiliation] OR "queensland"[Title/Abstract] OR "queensland"[Affiliation]) AND ("aborigin*"[Title/Abstract] OR "indigenous"[Title/Abstract])) OR "torres strait islander*"[Title/Abstract]) NOT "medline"[Filter]) OR ("Indigenous Peoples"[MeSH Terms] OR "indians, north american"[MeSH Terms] OR "Inuits"[MeSH Terms] OR ("first nation*"[Title/Abstract] OR "indigenous population*"[Title/Abstract] OR "tribe"[Title/Abstract] OR "tribal"[Title/Abstract] OR "Maori"[Title/Abstract] OR "Native American"[Title/Abstract] OR "inuit*"[Title/Abstract] OR "metis"[Title/Abstract]))) AND (((("Precision Medicine"[Mesh]) OR "Pharmacogenetics"[Mesh]) OR "Pharmacogenomic Variants"[Mesh]) OR ((((((("precision medicine"[Title/Abstract]) OR ("personalised medicine"[Title/Abstract])) OR ("personalized medicine"[Title/Abstract])) OR (genetic*[Title/Abstract])) OR (genomic*[Title/Abstract])) OR (pharmacogenetic*[Title/Abstract])) OR (pharmacogenomic*[Title/Abstract])))) AND (("Health"[Mesh]) OR (health[Title/Abstract]))
5. Search: (((((("australia"[MeSH Terms] OR "australia*"[Title/Abstract]) AND ("oceanic ancestry group"[MeSH Terms] OR "aborigin*"[Title/Abstract] OR "indigenous"[Text Word])) OR "torres strait islander*"[Title/Abstract]) AND "medline"[Filter]) OR (((("au"[Affiliation] OR "australia*"[Affiliation] OR "australia*"[Title/Abstract] OR "northern territory"[Title/Abstract] OR "northern territory"[Affiliation] OR "tasmania"[Title/Abstract] OR "tasmania"[Affiliation] OR "new south wales"[Title/Abstract] OR "new south wales"[Affiliation] OR "victoria"[Title/Abstract] OR "victoria"[Affiliation] OR "queensland"[Title/Abstract] OR "queensland"[Affiliation]) AND ("aborigin*"[Title/Abstract] OR "indigenous"[Title/Abstract])) OR "torres strait islander*"[Title/Abstract]) NOT "medline"[Filter]) OR ("Indigenous Peoples"[MeSH Terms] OR "indians, north american"[MeSH Terms] OR "Inuits"[MeSH Terms] OR ("first nation*"[Title/Abstract] OR "indigenous population*"[Title/Abstract] OR "tribe"[Title/Abstract] OR "tribal"[Title/Abstract] OR "Maori"[Title/Abstract] OR "Native American"[Title/Abstract] OR "inuit*"[Title/Abstract] OR "metis"[Title/Abstract]))) AND (((("Precision Medicine"[Mesh]) OR "Pharmacogenetics"[Mesh]) OR "Pharmacogenomic Variants"[Mesh]) OR ((((((("precision medicine"[Title/Abstract]) OR ("personalised medicine"[Title/Abstract])) OR ("personalized medicine"[Title/Abstract])) OR (genetic*[Title/Abstract])) OR (genomic*[Title/Abstract])) OR (pharmacogenetic*[Title/Abstract])) OR (pharmacogenomic*[Title/Abstract])))) AND (("Health"[Mesh]) OR (health[Title/Abstract]))

## Appendix 2 Completed PRISMA 2020 Checklist (Page et al. 2021)

### 2.1

**Table:**

Section and topic	Item #	Checklist item	Location where item is reported (pages)
*Title*			
Title	1	Identify the report as a systematic review	1
*Abstract*			
Abstract	2	See the PRISMA 2020 for Abstracts checklist	2
*Introduction*			
Rationale	3	Describe the rationale for the review in the context of existing knowledge	4
Objectives	4	Provide an explicit statement of the objective(s) or question(s) the review addresses	4
*Methods*			
Eligibility criteria	5	Specify the inclusion and exclusion criteria for the review and how studies were grouped for the syntheses	5–6
Information sources	6	Specify all databases, registers, websites, organizations, reference lists, and other sources searched or consulted to identify studies. Specify the date when each source was last searched or consulted	5
Search strategy	7	Present the full search strategies for all databases, registers, and websites including any filters and limits used	Appendix
Selection process	8	Specify the methods used to decide whether a study met the inclusion criteria of the review, including how many reviewers screened each record and each report retrieved, whether they worked independently, and if applicable, details of automation tools used in the process	6
Data collection process	9	Specify the methods used to collect data from reports, including how many reviewers collected data from each report, whether they worked independently, any processes for obtaining or confirming data from study investigators, and if applicable, details of automation tools used in the process	6
Data items	10a	List and define all outcomes for which data were sought. Specify whether all results that were compatible with each outcome domain in each study were sought (e.g., for all measures, time points, analyses), and if not, the methods used to decide which results to collect	7
	10b	List and define all other variables for which data were sought (e.g., participant and intervention characteristics, funding sources). Describe any assumptions made about any missing or unclear information	8
Study risk of bias assessment	11	Specify the methods used to assess risk of bias in the included studies, including details of the tool(s) used, how many reviewers assessed each study and whether they worked independently, and if applicable, details of automation tools used in the process	7
Effect measures	12	Specify for each outcome the effect measure(s) (e.g., risk ratio, mean difference) used in the synthesis or presentation of results	n/a
Synthesis methods	13a	Describe the processes used to decide which studies were eligible for each synthesis (e.g., tabulating the study intervention characteristics and comparing against the planned groups for each synthesis (item #5))	8
	13b	Describe any methods required to prepare the data for presentation or synthesis, such as handling of missing summary statistics, or data conversions	n/a
	13c	Describe any methods used to tabulate or visually display results of individual studies and syntheses	n/a

	13d	Describe any methods used to synthesize results and provide a rationale for the choice(s). If meta‐analysis was performed, describe the model(s), method(s) to identify the presence and extent of statistical heterogeneity, and software package(s) used	n/a
	13e	Describe any methods used to explore possible causes of heterogeneity among study results (e.g., subgroup analysis and meta‐regression)	n/a
13f	Describe any sensitivity analyses conducted to assess robustness of the synthesized results	n/a
Reporting bias assessment	14	Describe any methods used to assess risk of bias due to missing results in a synthesis (arising from reporting biases)	7
Certainty assessment	15	Describe any methods used to assess certainty (or confidence) in the body of evidence for an outcome	n/a
*Results*			
Study selection	16a	Describe the results of the search and selection process, from the number of records identified in the search to the number of studies included in the review, ideally using a flow diagram	8
	16b	Cite studies that might appear to meet the inclusion criteria, but which were excluded, and explain why they were excluded	7
Study characteristics	17	Cite each included study and present its characteristics	8
Risk of bias in studies	18	Present assessments of risk of bias for each included study	8
Results of individual studies	19	For all outcomes, present, for each study: (a) summary statistics for each group (where appropriate) and (b) an effect estimate and its precision (e.g., confidence/credible interval), ideally using structured tables or plots	8
Results of syntheses	20a	For each synthesis, briefly summarize the characteristics and risk of bias among contributing studies	30
	20b	Present results of all statistical syntheses conducted. If meta‐analysis was done, present for each the summary estimate and its precision (e.g., confidence/credible interval) and measures of statistical heterogeneity. If comparing groups, describe the direction of the effect	n/a
	20c	Present results of all investigations of possible causes of heterogeneity among study results	n/a
	20d	Present results of all sensitivity analyses conducted to assess the robustness of the synthesized results	n/a
Reporting biases	21	Present assessments of risk of bias due to missing results (arising from reporting biases) for each synthesis assessed	n/a
Certainty of evidence	22	Present assessments of certainty (or confidence) in the body of evidence for each outcome assessed	n/a
*Discussion*			
Discussion	23a	Provide a general interpretation of the results in the context of other evidence	41
	23b	Discuss any limitations of the evidence included in the review	42
	23c	Discuss any limitations of the review processes used	42
	23d	Discuss implications of the results for practice, policy, and future research	42
*Other information*			
Registration and protocol	24a	Provide registration information for the review, including register name and registration number, or state that the review was not registered	5
	24b	Indicate where the review protocol can be accessed, or state that a protocol was not prepared	5
	24c	Describe and explain any amendments to information provided at registration or in the protocol	5
Support	25	Describe sources of financial or non‐financial support for the review, and the role of the funders or sponsors in the review	43
Competing interests	26	Declare any competing interests of review authors	44
Availability of data, code, and other materials	27	Report which of the following are publicly available and where they can be found: template data collection forms; data extracted from included studies; data used for all analyses; analytic code; any other materials used in the review	43
